# Genomic analysis of plasma circulating tumor DNA in patients with heavily pretreated HER2 + metastatic breast cancer

**DOI:** 10.1038/s41598-023-35925-8

**Published:** 2023-06-19

**Authors:** Kyoungmin Lee, Jongwon Lee, Jungmin Choi, Sung Hoon Sim, Jeong Eun Kim, Min Hwan Kim, Yeon Hee Park, Jee Hyun Kim, Su-Jin Koh, Kyong Hwa Park, Myoung Joo Kang, Mi Sun Ahn, Kyoung Eun Lee, Hee-Jun Kim, Hee Kyung Ahn, Han Jo Kim, Keon Uk Park, In Hae Park

**Affiliations:** 1 Division of Oncology/Hematology, Department of Internal Medicine, Korea University Guro Hospital, Korea University College of Medicine, Seoul, Korea; 2 Brain Korea 21 Plus Project for Biomedical Science, Korea University College of Medicine, Seoul, Korea; 3 Department of Biomedical Sciences, Korea University College of Medicine, Seoul, Korea; 4 Department of Genetics, Yale University School of Medicine, New Haven, CT USA; 5 Center for Breast Cancer, National Cancer Center, Goyang, Korea; 6 Department of Oncology, Asan Medical Center, University of Ulsan College of Medicine, Seoul, Korea; 7 Division of Medical Oncology, Department of Internal Medicine, Yonsei Cancer Center, Yonsei University College of Medicine, Seoul, Korea; 8 Division of Hematology/Oncology, Department of Medicine, Samsung Medical Center, Sungkyunkwan University School of Medicine, Seoul, Korea; 9 Department of Internal Medicine, Seoul National University Bundang Hospital, Seoul National University College of Medicine, Seongnam, Korea; 10 Department of Hematology and Oncology, Ulsan University Hospital, Ulsan University College of Medicine, Ulsan, Korea; 11 Division of Oncology/Hematology, Department of Internal Medicine, Korea University Anam Hospital, Korea University College of Medicine, Seoul, Korea; 12 Division of Oncology, Department of Internal Medicine, Haeundae Paik Hospital, Inje University College of Medicine, Busan, Korea; 13 Department of Hematology-Oncology, Ajou University School of Medicine, Suwon, Korea; 14 Department of Hematology and Oncology, Ewha Womans University Hospital, Seoul, Korea; 15 Division of Hematology/Medical Oncology, Department of Internal Medicine, Chung-Ang University Hospital, Seoul, Korea; 16 Division of Medical Oncology, Department of Internal Medicine, Gachon University Gil Medical Center, Incheon, Korea; 17 Division of Oncology and Hematology, Department of Internal Medicine, Soonchunhyang University Cheonan Hospital, Cheonan, Korea; 18 Division of Hematology/Oncology, Department of Internal Medicine, Keimyung University Dongsan Hospital, Daegu, Korea

**Keywords:** Cancer, Breast cancer, Cancer genomics

## Abstract

We explored accumulated genomic alterations in patients with heavily treated HER2 + metastatic breast cancer enrolled in the KCSG BR18-14/KM10B trial. Targeted sequencing was performed with circulating tumor DNAs (ctDNAs) collected before the treatment of 92 patients. ctDNAs collected at the time of disease progression from seven patients who had a durable response for > 12 months were also analyzed. Sixty-five genes were identified as pathogenic alterations in 99 samples. The most frequently altered genes were *TP53* (n = 48), *PIKCA* (n = 21) and *ERBB3* (n = 19). *TP53* and *PIK3CA* mutations were significantly related with shorter progression free survival (PFS), and patients with a higher ctDNA fraction showed a worse PFS. The frequency of homologous recombination deficiency (HRD)-related gene mutations was higher than that in matched tumor tissues, and these mutations tended to be associated with shorter PFS. New pathogenic variants were found at the end of treatment in all seven patients, including *BRCA2, VHL, RAD50, RB1, BRIP1, ATM, FANCA,* and *PIK3CA* mutations. In conclusion, *TP53* and *PIK3CA* mutations, as well as a higher ctDNA fraction, were associated with worse PFS with trastuzumab and cytotoxic chemotherapy. The enrichment of HRD-related gene mutations and newly detected variants in ctDNA may be related to resistance to treatment.

## Introduction

HER2-positive breast carcinomas are known to exhibit HER2 amplification, which drives many oncogenic processes in cancer cells^1^. Currently, diverse anti-HER2 drugs have been developed, and are the standard treatment strategies for HER2-positive metastatic breast cancer (HER2 + MBC). Currently, dual anti-HER2 blockade antibodies (trastuzumab and pertuzumab) with cytotoxic chemotherapy and anti-HER2 antibody–drug conjugates (ADCs) [fam-trastuzumab-deruxtecan (T-Dxd) or trastuzumab emtansine (T-DM1)] are commonly recommended as first- or second-line therapies for HER2 + MBC^2,3^. For subsequent lines, anti-HER2 tyrosine kinase inhibitors, engineered antibodies, or the reuse of trastuzumab with different chemotherapy partners is suggested based on the concept of a continuum of anti-HER2 therapy^4^.

However, there is insufficient data and no consensus on how many times anti-HER2 therapy can be applied. Various acquired resistances accumulated through multiple treatments are expected in heavily pretreated patients, and tumor clonal evolution under therapeutic pressure may alter the genomic characteristics of primary tumor cells^5–7^. So far, there are few data regarding genetic alterations in heavily pretreated HER2 + MBC. New treatment options could be obtained for these populations by identifying the genetic alterations associated with treatment resistance.

Tissue-based next-generation sequencing (NGS) remains the gold standard for tumor genomic characterization^8^; however, the invasiveness of multiple tissue biopsies is a major hurdle, especially for heavily treated patients. Furthermore, the analysis of tumor tissue is limited in representing intra- and inter-tumor heterogeneity, which is the main cause of treatment resistance^9,10^. Blood-based liquid biopsy for detecting circulating tumor DNA (ctDNA) is an attractive alternative that can provide a heterogeneous picture of tumors and track dynamic genomic changes in an easy and safe manner^11,12^.

In this study, we explored the accumulated genomic alterations and their clinical significance through ctDNA analysis in patients with heavily treated HER2 + MBC using blood samples from patients who were enrolled in the KCSG BR18-14/KM10B trial.

## Results

### Overview of targeted sequencing of ctDNA results

In the present study, 100 peripheral blood samples were collected from 93 patients. The ctDNA data of 99 samples were included in the final analysis, as one sample that failed the Quality Control (QC) checks was excluded. Overall, 92 samples were obtained at the time of screening and paired end of-treatment (EOT) samples were obtained from seven patients. One patient failed to provide blood samples during screening, and only the EOT sample was collected. Of the 93 patients, 31 also provided FFPE tumor tissues (Supplementary Table S1). The clinical characteristics of the patient records included in the final dataset are presented in Table 1.

**Table 1:** Patients’ characteristics.

	N = 93
Age (years, median, range)	54 (20–70)
Menstruation status	
Premenopausal	32 (34.4%)
Postmenopausal	60 (64.5%)
Unknown	1 (1.1%)
HR status	
HR ( +)	53 (57.0%)
HR (−)	37 (39.8%)
Unknown	3 (3.2%)
De novo stage IV	29 (31.2%)
No. of prior chemotherapy regimens at metastatic setting* (median, range)	4 (2–11)
1–3	35 (38.0%)
> 3	57 (62.0%)
Unknown	1 (1.1%)
No. of prior anti-HER2 therapy at metastatic setting** (median, range)	3 (0–8)
1–3	71 (76.3%)
> 3	22 (23.7%)
Prior anti-HER2 agents	
Trastuzumab	90 (96.8%)
Pertuzumab	39 (41.9%)
Trastuzumab emtansine (T-DM1)	90 (96.8%)
Lapatinib	70 (75.3%)
Trastuzumab deruxtecan (T-Dxd)	9 (9.7%)

*HR* hormone receptor, *No.* number.

*Number of all administered chemotherapies regardless of anti-HER2 drugs in a metastatic setting.

**Number of anti-HER2 based treatments in a metastatic setting.

DNA isolated from plasma samples and tumor tissues was subjected to hybridization capture and targeted deep sequencing to detect somatic single-nucleotide variants (SNVs), small insertions and deletions (indels), and copy number alterations. The average concentration of ctDNA was 1.18 ng/μL (range 0.16–10.23 ng/μL) from 99 samples. A summary of targeted sequencing data is presented in Supplementary Tables S2 and S3. The mean target coverage for tissue samples was 809.25x (range, 176.7–1538.45x) and 4710x (range 1851–7161x) for plasma samples. Overall, a median of 96.4% and 99.6% of the target bases were satisfied, with a coverage of more than 100 × in tissue and plasma samples, respectively. This result indicated that we obtained adequate coverage of the target genes to detect genomic alterations with high sensitivity.

### Mutational landscape and prevalence of pathogenic variants in ctDNA

**Figure 1:**
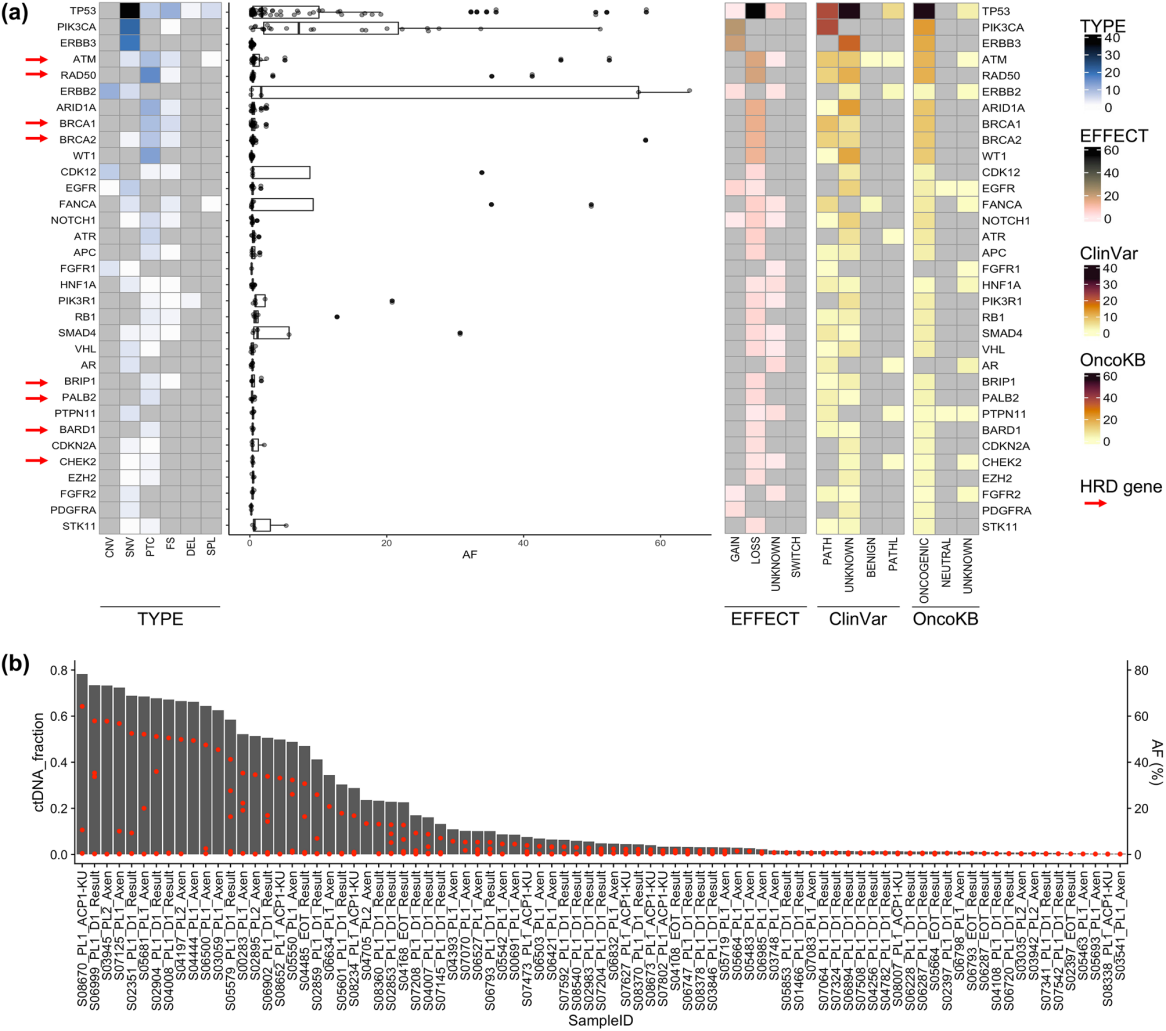
Genomic landscape of ctDNA in patients with metastatic breast cancer. (**a**) Mutational landscape represented by top 33 frequently mutated genes. More than two variants are identified on both sides of the figure. The heat map shows the number of variants of the top mutated genes referring to the mutation type (left), different functional classes, or pathogenicity (right). The distribution of allelic fractions for each variant is shown in a boxplot with jitter (center). Red arrows indicate the HRD genes. (**b**) ctDNA fraction (%, gray bars) calculated using pathogenic variants allelic fraction (Red dots) from the quantifiable 85 samples. HRD, homologous recombination deficiency. Variant annotations were defined as follows. Mutation type: CNV, Copy number variation; SNV, Missense_variant, splice_region_variant; PTC, stop_gained&splice_region_variant, stop_gained; FS: frameshift_variant, frameshift_variant&stop_gained, frameshift_variant&splice_region_variant DEL, conservative_inframe_deletion, disruptive_inframe_deletion, splice_donor_variant&disruptive_inframe_deletion&splice_region_variant&intron_variant; SPL, splice_acceptor_variant&intron_variant, splice_donor_variant&intron_variant, splice_region_variant, and intron_variant. EFFECT (functional classes)—GAIN, Gain_of_function, Likely Gain-of-function; LOSS, Loss of function; Likely Loss of function; SWITCH, Switch-of-function, Likely Switch-of-function; UNKNOWN, Unknown ClinVar-PATH, pathogenic, pathogenic/likely pathogenic; PATHL, Likelypathogenic; BENIGN, Benign; UNKNOWN, Conflicting_interpretations_of_pathogenicity, Uncertain_significance, not_provided. OncoKB-ONCOGENIC, oncogenic, likely oncogenic, predicted oncogenic; NEUTRAL, likely neutral; UNKNOWN, unknown; NA.

To examine the prevalence and relevance of any detected pathogenic variants in the ctDNA, a mutational landscape was generated from 99 ctDNA samples (Fig. 1A). After removing variants from the germline, blacklist, and clonal hematopoiesis, 336 pathogenic somatic alterations and 25 copy number alterations were identified in 65 genes (Supplementary Tables S4–S7). *TP53* was the most frequently altered gene (48 out of 99 samples), followed by *PIK3CA* (n = 21), *ERBB3* (n = 19), *ATM* (n = 17), *RAD50* (n = 16), *ERBB2* (n = 15), *ARID1A* (n = 12), and *BRCA2* (n = 11). This pattern is comparable to that observed in previous studies on breast cancer^13,14^.

The ctDNA fractions were further calculated to evaluate their utility as prognostic biomarkers in qualified samples. Of the 99 plasma samples, 85 were calculated using the maximum allelic fraction of the pathogenic variants, and the remaining 14 samples were excluded from the analysis because of the absence of a pathogenic variant. The median ctDNA fraction was 3.33%, and its distribution is shown in Supplementary Table S8 and Fig. 1B.

### Concordance of genetic alterations between ctDNAs and matched tumor tissues

**Figure 2:**
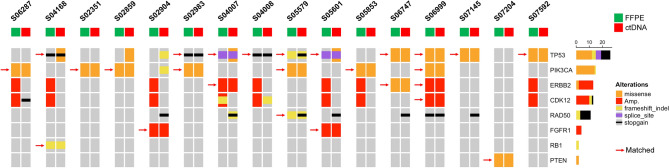
Oncoprint chart for top 8 genes from 16 patients who had shared 26 alterations in both tissue and plasma samples. Concordant gene alterations detected in both biopsies are indicated by red arrows. Green and red squares on the top represent tissue and plasma samples, respectively.

Of the 31 patients who had both plasma samples and matched tumor tissues, 135 genes were found to have somatic alterations in either sample. To determine the concordance between tissue and plasma samples, our analysis specifically concentrated on 61 genes that were covered by both the CancerSCAN and the Axen panel (Supplementary Table S9). The median time interval between tumor and plasma sample collection was 42 months (range 2–104 months). Sixteen of the 31 patients had 26 concordant alterations in both samples, including 18 SNVs, three indels, and five amplifications (Fig. 2 and Supplementary Table S10). The gene-level variant concordance rate between the tissue and ctDNA samples ranged from 73.8% (45/61) to 95.1% (58/61), including all alterations present or absent. *TP53* (10/31, 32.3%) showed the highest concordance rate at the gene level, followed by *PIK3CA* (7/31, 22.6%), ranged from 0% (0/31) to 32.3% (10/31), which is comparable to those reported in other breast cancer studies^15,16^.

We further analyzed several factors that could affect the concordance rate, such as the time of tumor tissue acquisition (primary or metastatic tissue), time interval between tissue and plasma sample collection, and ctDNA fraction. The concordance rate was not significantly different according to the time of tumor tissue acquisition or the time interval between tissue and plasma collection (data not shown). The ctDNA fraction of the concordant group (n = 16) was significantly higher than that of the discordant group (n = 15) (Supplementary Fig. S1 and Supplementary Table S8). This result suggests that a higher ctDNA fraction was a major factor in the concordance between plasma and tissue, which is consistent with the reports of previous studies^15,17^.

### Enrichment of HRD-related gene mutations in ctDNA

**Figure 3:**
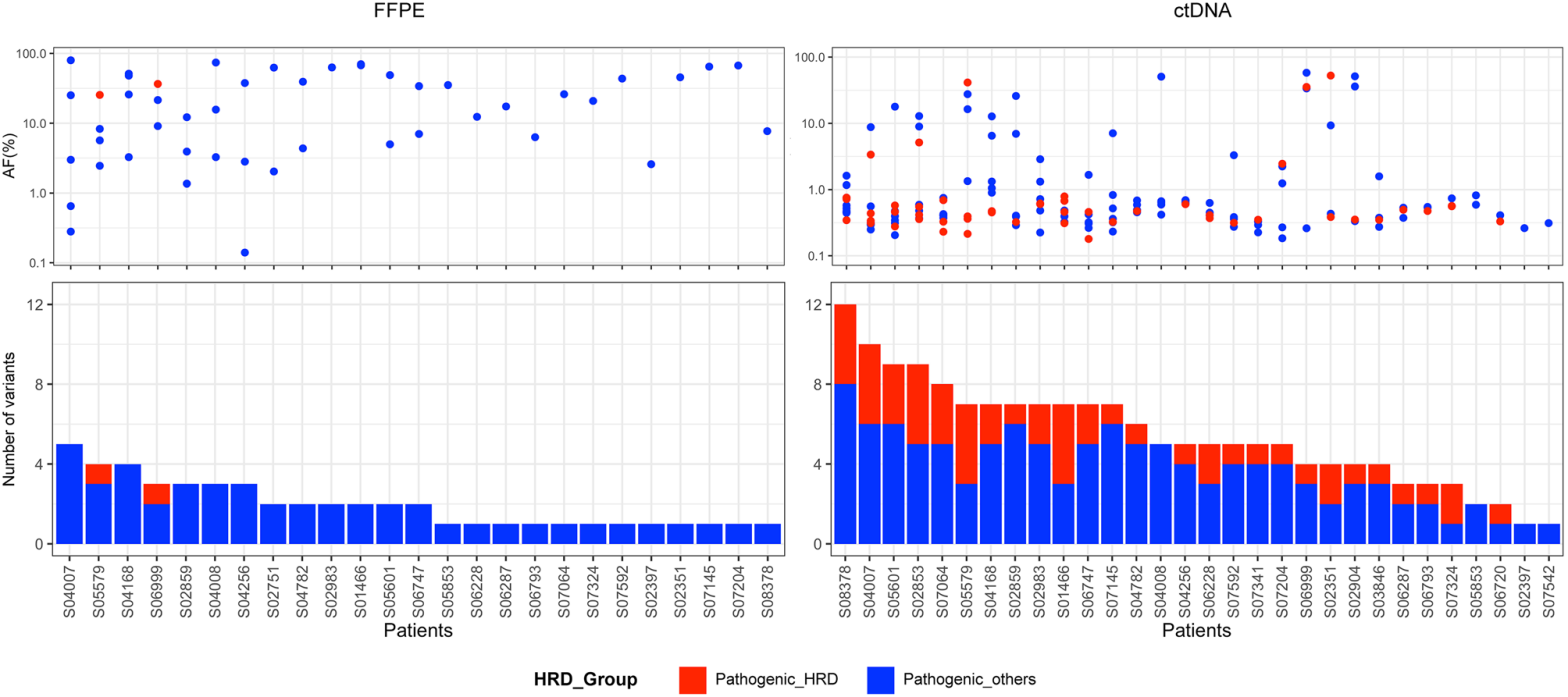
Enrichment of HRD-related gene mutation in ctDNA. Distribution of pathogenic variant allelic fraction and number of variants detected in FFPE or ctDNA samples. *HRD* homologous recombination deficiency, *FFPE* formalin fixed paraffin embedded.

We found that pathogenic alterations in HRD-related genes, including *ATM, RAD50, BRCA1/2, BRIP1, PALB2, BARD1* and *CHEK2* were enriched in ctDNA analysis data (Fig. 1). Therefore, we investigated whether the detection rate of HRD-related gene mutations differed between the tumor tissue and ctDNA from 31 pairs of samples. Most variants detected in ctDNA had AF as low as < 1%; however, the total number of pathogenic variants was higher in ctDNA. Among them, HRD-related gene mutations represented 7.1% (2 of 28 pathogenic alterations) of the tumor samples and 31.1% (51 of 164 pathogenic alterations) of the ctDNA samples (Fig. 3).

### Survival analyses according to the ctDNA genetic alterations

**Figure 4:**
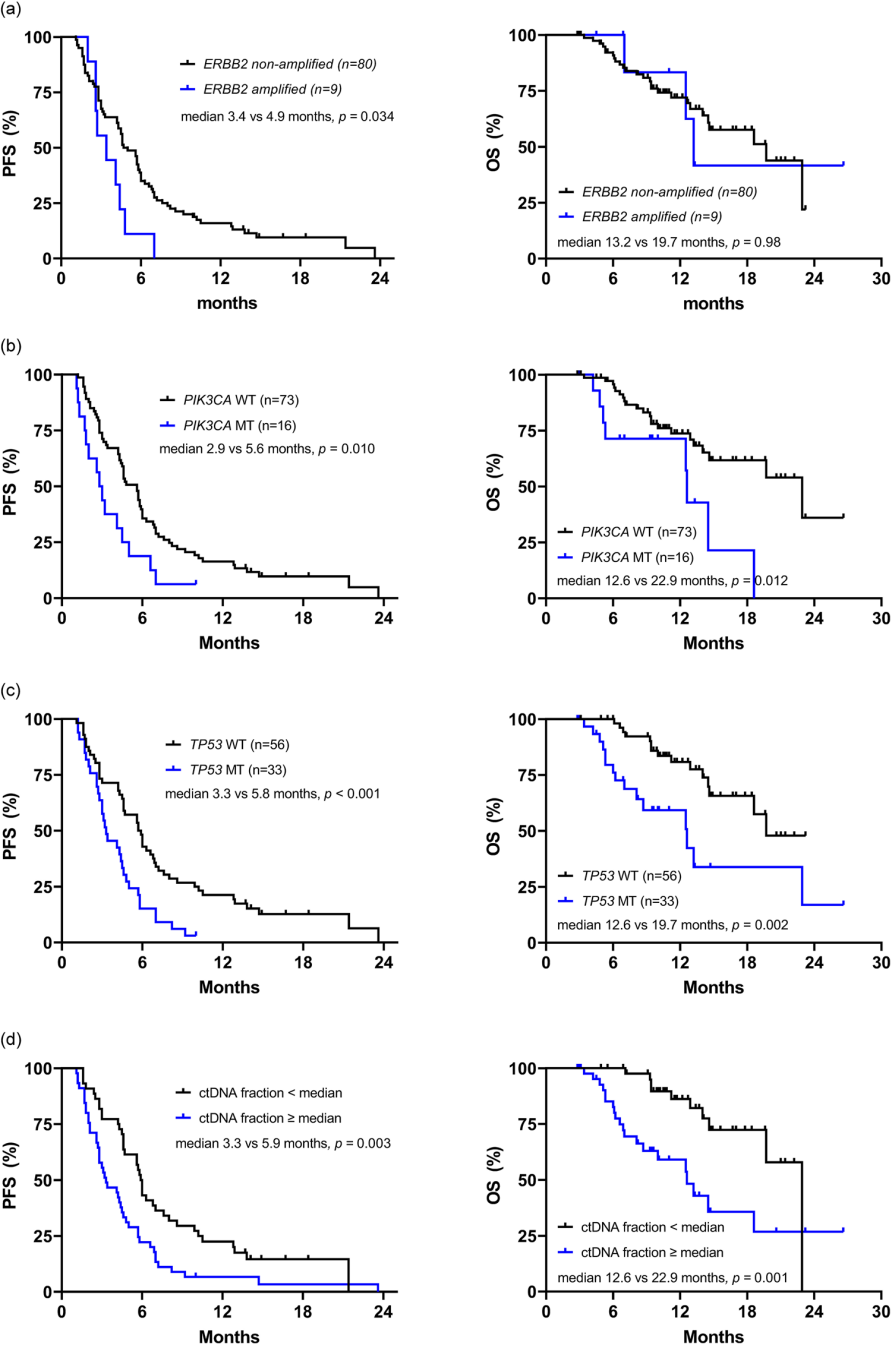
Kaplan–Meier estimates of progression free survival and overall survival on study treatment according to (**a**) *ERBB2* amplification, (**b**) *PIK3CA* mutation, (**c**) *TP53* mutation, and (**d**) ctDNA fraction. *PFS* progression free survival, *OS* overall survival.

Survival analysis was performed using data from 89 patients with survival information for the study treatment. With a median follow-up duration of 12.7 months (95% CI 10.3–15.1), the median PFS and OS of all 89 patients was 4.6 (95% CI 4.0–5.2) and 19.7 (95% CI 11.8–27.6) months, respectively. As shown in Fig. 4, *PIK3CA* (median 2.9 vs. 5.6 months, HR = 2.07, 95% CI 1.17–3.68, *p* = 0.010) and *TP53* (median 3.3 vs. 5.8 months, HR = 2.17, 95% CI 1.35–3.47, *p* < 0.001) mutations were significantly associated with shorter PFS. A higher ctDNA fraction (> 3.33% [median]) was also associated with a worse PFS (median 3.3 vs. 5.9 months, HR = 1.95, 95% CI 1.25–3.05, *p* = 0.003). *PIK3CA* mutation, *TP53* mutation, and higher ctDNA fraction were associated with poor OS (median 12.6 vs. 22.9 months, *p* = 0.012; 12.6 vs. 19.7 months, *p* = 0.002; 12.6 vs. 22.9 months, *p* = 0.001, respectively). The prognostic value of TP53 and PIK3CA mutations, as well as ctDNA fractions, remained consistent after adjusting for age (≤ 55 years vs > 55 years), number of prior anti-HER2 therapies (≤ 3 vs > 3), and presence of visceral metastasis (no vs yes) (Supplementary Table S11). *ERBB2* amplification was detected in only 15 patients, and patients with *ERBB2* amplification had worse PFS (median 3.4 vs. 4.9 months, HR = 2.11, 95% CI 1.04–4.31, *p* = 0.034). Of note, patients with HRD gene mutations tended to have shorter PFS, although this was not statistically significant (median 3.2 vs. 4.9 months, HR = 1.66, 95% CI 0.91–3.03, *p* = 0.09) (Supplementary Fig. S2).

### Post-treatment clonal evolutions detected by paired sample analyses

**Figure 5:**
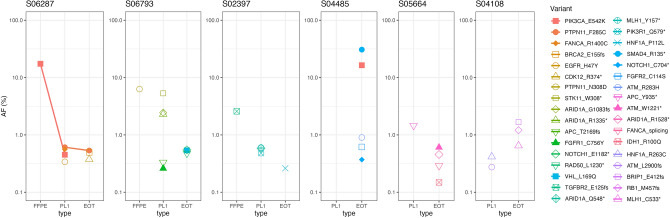
Changes in allelic fraction of pathogenic variants detected in FFPE or serial ctDNA samples. *PL1* ctDNA at initial treatment, *EOT* ctDNA at end of treatment.

To identify the potential mechanisms of treatment resistance, we examined pathogenic variants from six patients who had paired ctDNA (Initial and EOT) (Fig. 5). These six patients had PFS longer than 12 months; therefore, we hoped to identify the mechanisms of acquired resistance from the analysis of samples from these patients. Three of the patients (S06287, S06793, and S02397) also had tumor tissue samples. For S06287, the *PIK3CA_E542K* mutation was detected in both the tumor tissue and initial ctDNA. However, this was not observed in the EOT sample, instead revealing previously undetected *BRCA2* and *CDK12* mutations. In S04485, the *PIK3CA_E542K* mutation was not initially found; however, it was detected with high AF at EOT. In addition, pathogenic HRD-related gene mutations were found in EOT samples, except for S02397, including *BRCA2, RAD50, ATM, FANCA*, and *BRIP1*.

## Discussion

HER2 overexpression in HER2 + breast cancer is an important oncogenic driver and therapeutic target; however, its genetic heterogeneity is known to be an important resistance mechanism to anti-HER2 therapy^18–20^. Moreover, due to tumor evolution and treatment pressure, genomic alterations in metastatic breast cancer can differ substantially from those in the primary tumor and become more disparate after multiple lines of chemotherapy^7,21^. In this study, we demonstrated the genomic landscape of ctDNA in patients with HER2 + MBC whose disease progressed after multiple lines of standard HER2-directed chemotherapy.

Consistent with previous studies^22,23^, *TP53* and *PIK3CA* mutations were the two most altered genes. It is well known that the frequency of *TP53* mutation is especially high in HER2 + breast cancer, and *TP53* mutation per se is associated with early onset breast cancer and poor prognosis^24^. Recently, Liu et al. demonstrated the potential of *TP53* mutation as a biomarker for predicting the efficacy of anti-HER2 therapies, including antibody-based drugs and TKIs, through ctDNA analysis^25^. In our study, patients with *TP53* mutations had worse PFS and OS. Although the predictive role of *TP53* mutations cannot be justified due to the limitation of the single-arm study design, the development of targeting *TP53* mutations seems necessary for patients with worse prognosis. Aberrant activation of PI3K is also a well-known oncogenic driver in breast cancer, including the HER2 + subtype, and is one of the major resistance mechanisms in anti-HER2 therapy^26^. A previous study showed that patients with HER2 + breast cancer with an activating *PIK3CA* mutation had lower pathologic complete response (pCR) rates and shorter PFS with palliative HER2-targeted therapy^27^. *PIK3CA* mutations in plasma ctDNA also predict survival and treatment outcomes in various types of advances cancers^28^, and a recent meta-analysis validated this finding in breast cancer^29^. In our study, patients with *PIK3CA* mutations in ctDNA showed a low treatment response and poor survival outcomes. In addition, *PIK3CA* pathogenic mutation with high AF was detected in the ctDNA when the disease progressed in one patient, indicating a possible therapeutic target for this patient. Currently, many clinical trials using PIK3CA inhibitors with anti-HER2 therapy are ongoing, and these studies are expected to inform the role of PIK3CA inhibitors in HER2 + breast cancer^26^. Beyond detecting single gene alterations, we calculated the ctDNA fraction and demonstrated its possible role as a prognostic biomarker in HER2 + MBC. ctDNA is assumed to reflect the tumor burden and has been suggested as a tool for prognostication and follow-up in patients with advanced cancers^30,31^. Several studies have shown the prognostic ability of ctDNA fractions in patients with MBC^22,23^, and a recent study advocated its role as a pragmatic, independent prognostic biomarker across the four most common advanced cancer types, including MBC, in real-world settings^32^. Moreover, although not addressed in our study, several studies have found that early on-treatment ctDNA dynamics were a surrogate for treatment outcomes^33,34^. However, many issues need to be addressed before applying ctDNA fractions in actual clinical practice, such as panel sensitivity, standard formula for calculating ctDNA fraction, and reliable cut-off values.

Interestingly, our findings showed that mutations of HRD-related genes were more frequent in ctDNA than in tumor tissue, and patients with HRD-related gene mutations tended to have shorter PFS. Additionally, various pathogenic HRD gene mutations have been detected in EOT samples collected during disease progression. There is limited data suggesting the accumulation of alterations in HRD-related genes during multiple chemotherapy cycles. However, considering the functional aspects of HRD-related genes, this enrichment suggests the presence of tumor cells that can survive despite impaired DNA repair mechanisms caused by prior anticancer therapy. And these tumor cells are likely to be resistant to subsequent treatments. Further studies are needed to determine whether they can be targeted.

In our study, *ERBB2* (*HER2*) amplification was detected in 15 patients (16%) and was reanalyzed and confirmed by droplet digital PCR (data not shown). The positive rate of HER2 in ctDNA is significantly lower than the previously reported 33–96% for metastatic HER2 + breast cancer and 9–31% for early breast cancer^35,36^. The low detection of ERBB2 in ctDNA could potentially be attributed to the previous anti-HER2 treatment effects. However, it would be challenging to draw definitive conclusions without confirming the presence of ERBB2 amplification in corresponding tumor cells. Our results reveal an intriguing association between *ERBB2* amplification in ctDNA and poor PFS. Assuming that low levels of *ERBB2* in ctDNA resulted from prior anti-HER2 treatment, the persistent high detection of *ERBB2* in ctDNA suggests a potential resistance to anti-HER2 therapy. This observation aligns with the results of the phase III KATHERINE study where high *HER2* RNA expression in residual tumors after trastuzumab containing neoadjuvant treatment was associated with worse outcomes in the trastuzumab adjuvant treatment group^37^. Additionally, the CALGB40601 study reported worse relapse free survival associated with the HER2 enriched subtype in residual disease following neoadjuvant treatment including trastuzumab ± lapatinib^38^. Considering these findings, the persistent high detection of *ERBB2* amplification in ctDNA may serve as a potential biomarker of anti-HER2 therapy, warranting further validation in future studies. Our study was conducted as a translational study parallel to a prospective clinical trial but had some limitations. First, no tumor tissue collected at the same time point could be compared with the ctDNA analysis results. Therefore, newly detected variants, including *ERBB2* amplification, in ctDNA could not be confirmed. Second, there was a considerable time interval between tissue and plasma sample collection. The relatively low concordance may be due to tumor evolution occurring over such a long time, but this needs to be verified further. Finally, we did not collect serial follow-up ctDNA samples from all patients. Therefore, data on the association between ctDNA dynamics and treatment effect could not be obtained.

In conclusion, *TP53* and *PIK3CA* mutation and a higher ctDNA fraction were associated with worse PFS and OS in patients with heavily treated HER2 + MBC. Accumulation of mutations in HRD-related genes was noted in the ctDNA analysis and was related to shorter PFS. ctDNA analysis can be useful for predicting the efficacy of anti-HER2 treatment and patient prognosis, even in heavily pretreated populations. It can also provide valuable information regarding new therapeutic targets for these patients.

## Methods

### Patient cohort and sample collection

The KCSG BR18-14/KM10B trial was a multicenter, single-arm, phase 2 study that investigated the efficacy and safety of a trastuzumab biosimilar, Herzuma®, in combination with the treatment of physician’s choice (TPC) in patients with HER2 + MBC who had failed 2 or more HER2 directed chemotherapies^39^. The median PFS and OS were 4.6 months (95% CI 2.8–7.2) and 18.6 months (95% CI 14.4-not reached), respectively. For exploratory biomarker analysis, blood samples (pre-treatment/disease progression) and/or formalin-fixed paraffin-embedded (FFPE) tumor tissues (primary or metastatic) were obtained from all participants (n = 110). Among them, 17 patients who failed to provide ctDNA were excluded for this analysis. For the blood samples, 10 mL of whole blood was collected with Cell-Free DNA BCT® for ctDNA preparation. Written informed consent was obtained from each patient prior to performing all study procedures. This study was approved by the Institutional Review Board of the Korea University Guro Hospital (IRB number 2019GR0420) and conducted in accordance with the guidelines for Good Clinical Practice and the Declaration of Helsinki.

### NGS and genomic analysis

Targeted sequencing of tumor tissues and ctDNAs was carried out with the K-MASTER project, a Korean solid cancer genome analysis research project^40^. Targeted sequencing using tumor tissues were performed using CancerSCAN™ (Samsung Genome Institute, Seoul, Korea) which encompasses the exons of 396 human cancer related genes (Supplementary Table S12). In the case of ctDNA analysis, we utilized the K-Master Axen™ Cancer Panel (Macrogen, Seoul, Korea) targeting the exons of 89 human cancer related genes (Supplementary Table S13)^41^. DNA input for generate sequencing library was 200 ng for FFPE and 10 ng for ctDNA samples. Paired-end 150 bp reads were generated on a HiSeq2500 or NovaSeq6000 sequencing system (Illumina, San Diego, CA, USA). Detailed methods for targeted sequencing and data processing were the same as previous reports except removing frequently detected variants that are likely to be alignment artifacts (blacklists) and germline variants^40^.

ClinVar (Pathogenic or Likely-pathogenic) or OncoKB (Oncogenic or Likely-oncogenic) database were referred to identify the pathogenic mutations. To exclude the influence of clonal hematopoiesis related variants, hematopoietic-associated gene mutations were further investigated via literature review^42,43^ and COSMIC database.

Concordance was defined at the single-gene level as detection of an identical alteration, as previously described^16^. The ctDNA fraction was estimated based on the allelic fraction of somatic mutations as described^44^ but relying on a modified approach as measured by the maximum somatic mutant allele fraction (MSAF)^14^. Homologous recombination deficiency (HRD) was defined as germline or somatic pathogenic alterations of the following 11 genes: *BRCA1, BRCA2, ATM, BARD1, BRIP1, CHEK2, PALB2, RAD51B, RAD51C, RAD51D*, and *RAD50*, as previous reports^45–47^.

### Statistical analysis

Patient demographics and genomic alterations were summarized using descriptive statistics. The Wilcoxon signed-rank test was used to evaluate statistical differences in ctDNA fractions between the concordant and discordant groups. To determine the clinical significance of specific genetic alterations, the progression-free survival (PFS) and overall survival (OS) of the study treatment were analyzed. Survival curves were generated with GraphPad Prism 5 (GraphPad Software, San Diego, CA, USA) using the Kaplan–Meier method and compared using the log-rank test. In the survival analysis, a mutation with an allele frequency (AF) of ≥ 1% was defined as positive^48,49^. All other statistical analyses were performed using R version 4.0.3 (R Foundation for Statistical Computing, Vienna, Austria), and statistical significance was two-tailed, with a significance set at *P* ≤ 0.05.

### Ethics approval statement

This study was approved by the Institutional Review Board of the Korea University Guro Hospital (IRB number 2019GR0420) and conducted in accordance with the guidelines for Good Clinical Practice and the Declaration of Helsinki.

## Supplementary Information

Supplementary Information 1.

Supplementary Information 2.

## Data Availability

The data generated in this study are available within the article and its supplementary data files, and further inquiries can be directed to the corresponding authors.
