# Supplementary material for: Genomic analysis of plasma circulating tumor DNA in patients with heavily pretreated HER2 + metastatic breast cancer
Source: Sci Rep. 2023 Jun 19;13:9928. doi: 10.1038/s41598-023-35925-8 (PMC10279711; doi:10.1038/s41598-023-35925-8)

Figure S1. Comparison of several factors could affect to concordance of genetic alterations.
(a) The boxplots of ctDNA fraction between concordant and discordant group.
(b) The sample collection time interval between concordant and discordant group.

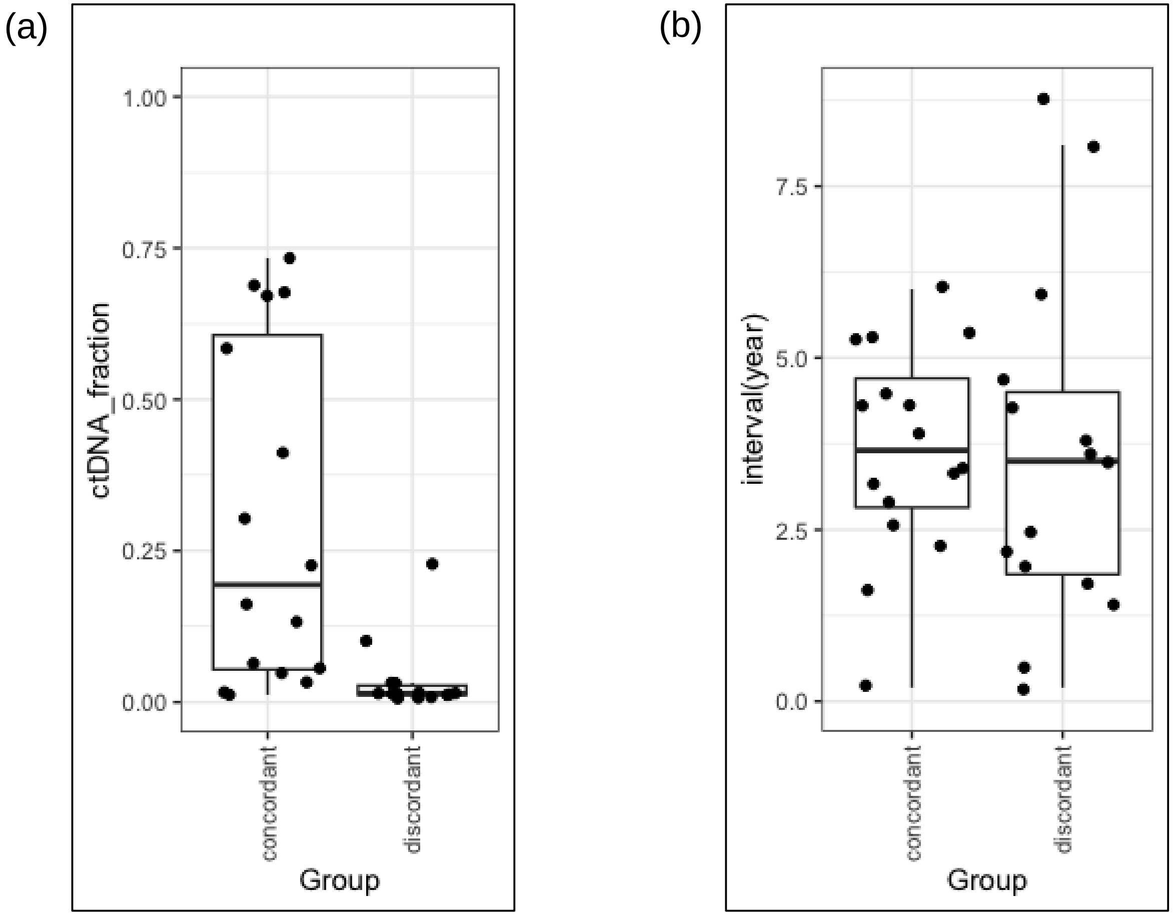

Wilcoxon test p-value = 1.195e-4

wilcoxon test p-value = 0.553

Figure S2. The Kaplan-Meier estimates of progression free survival on study treatment according to HRD-related gene mutation.
PFS, progression free survival; HRD, homologous recombination deficiency

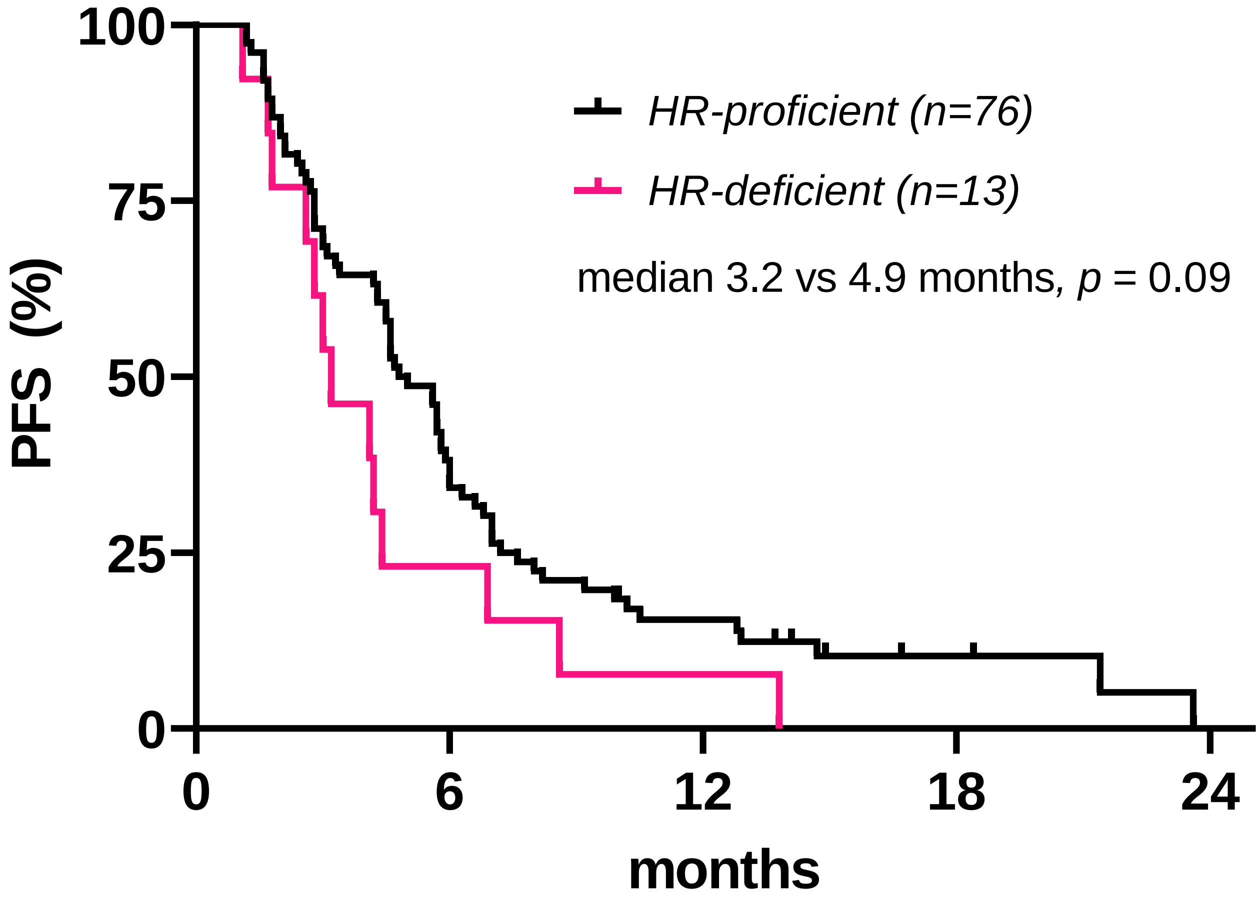

Supplement: Supplementary file 2 — Supplementary Information 2. [file 41598_2023_35925_MOESM2_ESM.pdf]
